# Water-soluble phthalocyanine photosensitizers for photodynamic therapy

**DOI:** 10.55730/1300-0527.3583

**Published:** 2023-09-26

**Authors:** İpek ÖMEROĞLU, Mahmut DURMUŞ

**Affiliations:** Department of Chemistry, Faculty of Science, Gebze Technical University, Kocaeli, Turkiye

**Keywords:** Phthalocyanine, water-soluble, photodynamic therapy, ionic or nonionic, photosensitizer, singlet oxygen

## Abstract

Photodynamic therapy (PDT) is based on a photochemical reaction that is started when a photosensitizing process is activated by the light and results in the death of tumor cells. Solubility is crucial in PDT applications to investigate the physical and chemical characteristics of phthalocyanines, but, unfortunately, most phthalocyanines show limited solubility especially in water. To increase the solubility of phthalocyanines in polar solvents and water, ionic groups such as -SO_3_^−^, -NR_3_^+^, -COO^−^, and nonionic groups such as polyoxy chains are frequently added to the peripheral or nonperipheral positions of the phthalocyanine framework. Since water-solubility and NIR-absorbing properties are essential for efficient PDT activation, studies have been focused on the synthesis of these types of phthalocyanine derivatives. This review focuses on the photophysical, photochemical, and some in vitro or in vivo studies of the recently published ionic and nonionic phthalocyanine-mediated photosensitizers carried out in the last five years. This review will have positive contributions to future studies on phthalocyanine chemistry and their PDT applications as well as photochemistry.

## 1. Introduction

Cancer, one of the most threatening diseases to human health worldwide, causes the deaths of millions of people, despite the research carried out in recent years. Despite the success of traditional treatment methods such as surgery, immunotherapy, radiotherapy, or chemotherapy, these treatments have some serious side effects. Therefore, scientists are focused on the improvement of new therapeutic treatment methods [1,2]. Photodynamic therapy (PDT) is accepted as a suitable treatment method to achieve rapid therapeutic results and reduce drug resistance in tumor cells [3]. In addition, PDT is an alternative treatment process for malign tumors that relies on photosensitizers to transfer light energy to reactive oxygen species (ROS) to induce cell apoptosis and tissue damage [4]. PDT performs its anticancer effect directly through cell death, damage to the vasculature, and activation of the immune system [5]. Cellular damage is due to the action of three components: a harmless photosensitizer, suitable light irradiation, and molecular oxygen [6]. This modern and appealing process depends on the usage of a combination of a photosensitizer, long wavelength light (620–690 nm), and molecular oxygen to selectively destroy or damage localized cancer tumors [7].

In Figure, the two photochemical mechanisms of PDT called Type I and Type II are schematically shown in the Jablonski diagram [8]. Reactive oxygen species (ROS) such as superoxide (O_2_^•^), hydroxyl radical (OH^•^), and hydrogen peroxide (H_2_O_2_) [9] are known as Type I photochemical mechanisms. When the photosensitizer transmits its energy to biomolecules in the excited ternary state, hydrogen or electron is transmitted to the free radicals. As a result of this transfer, photosensitizer and anion radicals of the substrate are formed between the photosensitizer and cancerous tissue. When electrons and oxygen molecules interact with each other, this process leads to the production of ROS. The other mechanism of PDT, called Type II, is defined as the formation of singlet molecular oxygen (^1^O_2_), which is the most important in the destruction of cancer cells. When the photosensitizer is exposed to electronic excitation from the ground state (S_0_) to the excited singlet (S_1_) state, the S_1_ state is highly unstable, and the electron rapidly transitions to the longer-lived excited triplet (T_1_) state via intersystem crossing. The photosensitizer then transfers its energy to molecular oxygen to form singlet oxygen (^1^O_2_) which is a highly reactive oxygen species. Singlet oxygen can cause the death of bacteria, fungi, or tumor cells in a specific location [10].

For a photosensitizer to be considered suitable for a Type I or Type II process, it should contain the following criteria:

- Powerful red or near-infrared absorption to authorize deep penetration of light into tissue,
- Insignificant dark toxicity and small side effects,
- High cytotoxicity under light irradiation,
- Good solubility (The solubility in aqueous solutions is very important for PDT applications. On the other hand, solvents with low cytotoxicity such as DMSO are very important as an alternative to aqueous solutions.),
- Stability in media,
- Preferred gathering in cancerous tissue [11].

Phthalocyanines are macrocyclic compounds formed by the coordination of four iminoisoindoline units. They are thermally, physically, and chemically consistent compounds due to their delocalized π electron systems. They are used in many applications such as semiconductors [12], dye-based solar cells [13], electrochromic systems [14], molecular electronics [15], liquid crystals [16], data storage materials [17], laser dyes [18], chemical sensors [19], catalysts [20], and PDT for cancer treatment [21] due to their excellent electrochemical, thermal, and optical properties. Since phthalocyanines have a very similar structure to that of porphyrins and have suitable properties for ideal photosensitizers, they are known as a class of second-generation photosensitizers [22,23]. Moreover, these compounds are used as photosensitizers in PDT because they show strong light absorption between 600 and 800 nm in the electronic spectrum, do not show toxicity in the absence of light, and destroy tumor tissues by producing high singlet oxygen or radicals [24]. On the other hand, the solubility problems of the phthalocyanines lead to a decrease in the absorption coefficient, which is important for singlet oxygen production. To solve this problem, peripheral/nonperipheral and/or axial substitution can be added to the phthalocyanine ring or metal ion to reduce aggregation and increase solubility [25,26]. The substituents placed on the skeleton and the metal atoms located in the cavity of the phthalocyanine ring can change the photophysical and photochemical properties of phthalocyanine compounds [27]. Studies have shown that diamagnetic metals increase singlet oxygen production and photoactivity compared to paramagnetic metals. Zinc, silicon, indium, gallium, and aluminum phthalocyanines, which have diamagnetic center atoms, are widely used as photosensitizers in photodynamic therapy [23]. These metal atoms promote a high quantum yield of excited triplet state in the PDT application and indicate high singlet oxygen yield [28,29]. Despite their strong properties, their poor solubility in water and their aggregation in polar environments complicate the applicability of these compounds in PDT [30]. Many photosensitizers are used for PDT, including those approved for humans; they tend to form aggregations, resulting in low water solubility. The aggregation and low water solubility render photosensitizers inactive in PDT, significantly limiting their in vivo studies. For these reasons, water-soluble and NIR (near-IR)-absorbing photosensitizers are essential for efficient PDT [31]. Studies have focused on the synthesis of water-soluble phthalocyanines because these compounds are suitable for different applications such as antioxidant, antibacterial, DNA binding/cleavage, enzyme inhibition, cytotoxic/phototoxic anticancer, and PDT activities [32]. Solubility is very important for the examination of the physical and chemical properties of phthalocyanines, but they indicate restricted solubility in most solvents. Substitutions at peripheral or nonperipheral positions of the macrocycle for these compounds improve their solubility. In addition, the solubility of phthalocyanines in organic solvents and water is often enhanced by the addition of polar or ionic groups (-SO_3_^−^, -NR_3_^+^, -COO^−^) at peripheral or nonperipheral positions [33,34]. Moreover, nonionic phthalocyanines contain polyethylene glycol/polyhydroxy, and carbohydrates can also gain their water solubility [35].

This review focuses on the photophysical, photochemical, in vitro, and in vivo studies of the water-soluble phthalocyanine compounds containing ionic or nonionic groups for PDT applications in the last five years. The results of these studies for different water-soluble phthalocyanine compounds are summarized. The photophysical, photochemical, and in vitro or in vivo biological properties of the phthalocyanine compounds are given as tables and these properties are compared with each other according to the central atom, the nature, and number of substituted groups. In the studies on the water-soluble phthalocyanines carried out in the last five years, the highest singlet oxygen quantum yield was obtained as 0.93 in H2O + Triton X-100 solution for the phthalocyanine derivative bearing indium (III) as a central metal atom and tetra quaternized 7-oxy-4-(pyridine-3-yl) groups on the peripheral positions of the phthalocyanine framework [58].

## 2. Ionic water-soluble phthalocyanines

Ionic water-soluble phthalocyanine compounds are classified into three groups: anionic, cationic, and zwitterionic [35,36].

Anionic groups such as carboxylate (-COO^−^), sulfonate (-SO_3_^−^), and phosphorus-based functions are generally used to bring water solubility to phthalocyanines. These groups are added directly to the phthalocyanine ring or by linker atoms such as oxygen, sulfur, or nitrogen. Phthalocyanines bearing sulfonate or sulfonic acid groups are synthesized by direct sulfonation of the phthalocyanine macrocycle or addition of the sulfonate-bearing substituted groups on the peripheral, nonperipheral, or axial positions [33]. Although both anionic and cationic phthalocyanines provide solubility in water, there are significant differences between anionic (containing carboxy or sulfo groups) and cationic (containing quaternary ammonium groups) phthalocyanines. In vitro studies show that cationic phthalocyanines are generally much more active in PDT applications than anionic phthalocyanines [37]. This observed behavior is explained by the effect of better ionization, subcellular localization and relocalization following radiation exposure, interactions with biomembranes, and differential binding to serum proteins of the cationic phthalocyanines [38].

Cationic groups are synthesized by the quaternization of the phthalocyanines on the aliphatic or aromatic nitrogen atoms in the substituted groups [35]. Metallophthalocyanines, especially silicon phthalocyanine derivatives containing cationic substituents, have some advantages over neutral and anionic substituents, such as improving water solubility, being a more efficient PDT agent by preventing aggregation, improving cell uptake, and selectively localizing in cell mitochondria [39].

Phthalocyanine compounds that carry anionic and cationic charges on the same molecule are called zwitterionic compounds. 1,3-propanesultone is usually used to obtain water-soluble zwitterionic phthalocyanine, and the sulfonate group is obtained by opening of 1,3-propanesultane ring [40].

## 3. Nonionic water-soluble phthalocyanines

Although nonionic water-soluble phthalocyanines are scarce compared to ionic phthalocyanines, they attract attention because they can interact with the cell membrane and components of biological fluids differently from ionic species. Therefore, phthalocyanine compounds containing nonionic groups such as carbohydrate or polyoxy are synthesized to obtain water-soluble phthalocyanines [41].

The functionalization of phthalocyanine compounds with polyethylene glycol is becoming progressively significant as it contributes positively to the chemical inertness, biocompatibility, improved serum life, and tumor cell accumulation of the compounds [42]. The addition of the hydrophilic moieties to the hydrophobic phthalocyanine core increases solubility and forms amphiphilic molecules, which is a desirable property for an effective photosensitizer [43]. The increasing number and length of polyoxy chains enhance the water solubility of the phthalocyanine compounds [35]. Moreover, nonionic polyhydroxylated groups are used to obtain water-soluble phthalocyanines [41,44].

Carbohydrates are used as biocompatible substituents that increase the water solubility of the phthalocyanines, which provides a potential for selective recognition by targeted cancer cells [45,46]. It has been determined that sugar-containing phthalocyanine compounds improve PDT efficiency due to increased glycolysis levels and overexpression of sugar carrier proteins in various human cancers, and glycosylated phthalocyanines are ideal photosensitizers [47].

## 4. Photophysical and photochemical studies of water-soluble phthalocyanines

The determination of the photophysical (fluorescence quantum yields and lifetimes) and photochemical (single oxygen yields) properties of the phthalocyanines are very important for photosensitizers in PDT applications.

Fluorescence properties such as fluorescence quantum yield (Φ_F_) and fluorescence lifetime (τ_F_) play an important role in PDT applications for visualizing of the photosensitizers in the body [48]. One of the most important factors in the evaluation of PDT is the singlet oxygen production. The highly effective reactivity of singlet oxygen can cause severe damage to biological systems such as DNA and RNA, resulting in cell death. When enough singlet oxygen is produced during the energy transfer from the photosensitizer to the oxygen molecule, effective cell death can be obtained [49].

### 4.1. Fluorescence quantum yields (Φ_F_) and lifetimes (τ_F_)

Fluorescence is the phenomenon where light is emitted by a molecule that has absorbed light. The fluorescence quantum yield (Φ_F_) is a quantification of the performance of the fluorescence process. Fluorescence lifetime (τ_F_) attributes to the average time a molecule remains in the excited state before fluorescence and is closely related to fluorescence quantum yield. It is very important to determine these values since an ideal photosensitizer should have a certain fluorescence quantum efficiency and fluorescence lifetime for the determination of the photosensitizers in the body [50]. The optimum fluorescence quantum yield is an essential component for the photosensitizer due to its deposition and subsequent evacuation from the tissue. Fluorescent emission may be utilized to monitor the use of photosensitizers in the body [51].

Fluorescence quantum yield (Φ_F_) is determined by using equation (1):


(1) $$\Phi_{\text{F}}=\Phi_{\text{F }(\text{Std})}\frac{F.A_{Std}.n^{2}}{F_{Std}.A.n_{Std}^{2}}$$

where F and F_Std_ are the areas under the fluorescence curves of the sample and the standard, respectively. A and A_Std_ are the absorbances of the sample and standard at the excitation wavelength, and n and n_Std_ are the refractive indices of the solvents used for the sample and standard, respectively [52].

Fluorescence lifetime (τ_F_), which is directly concerned with fluorescence quantum yield (Φ_F_), refers to the average time a molecule stays in its excited state before fluorescing. When a compound has a longer lifetime, it has a higher fluorescence quantum yield [53].

Temperature, molecular structure, and solvent properties, including polarity, viscosity, refractive index, and the presence of heavy atoms in the solvent molecule, can all have an impact on the fluorescence quantum yield values [54]. The fluorescence lifetime of a photosensitizer is also affected by a variety of parameters including internal conversion, intersystem migration, aggregation, and solvent [55].

### 4.2. Singlet oxygen quantum yields (Φ_Δ_)

Since PDT is a treatment method based on the destruction of cancer cells by singlet oxygen [56], an efficient photosensitizer must produce effective singlet oxygen to be used in the treatment of cancer with photodynamic therapy. The amount of the production of singlet oxygen is given as singlet oxygen quantum yield (Φ_Δ_) [57].

Singlet oxygen quantum yield (Φ_Δ_) is described as the quenching of absorption for a singlet oxygen quenching compound. The decrease in the quencher absorption at 417 nm for 1,3-diphenylisobenzofuran (DPBF) in organic solutions and 380 nm for 9,10-antracenediyl-bis(methylene)dimalonoic acid (ADMA) in aqueous media are monitored by UV-Vis spectrophotometry. Singlet oxygen quantum yield (Φ_Δ_) is determined by using equation (2):


(2) $$\Phi_{\Delta }=\Phi_{\Delta }^{Std}\frac{R.I_{abs}^{Std}}{R^{Std}.I_{abs}}$$

where $\Phi_{\Delta }^{Std}$ is the singlet oxygen quantum yield of the standard, R and R_Std_ are the quencher’s photobleaching rates in the presence of the sample and standard, respectively. I_abs_ and I_Std_ abs are the rates of light absorption by the sample and standard, respectively [53].

Photophysical and photochemical properties of ionic and nonionic phthalocyanine compounds containing different groups are given in Table 1. When the studies in the last five years were examined, the highest singlet oxygen yield was determined as 0.93 in H_2_O + Triton X-100 solution for indium (III) phthalocyanine compound containing peripheral quaternized 7-oxy-4-(pyridine-3-yl)coumarin groups [58]. The nonperipheral substituted zinc(II) phthalocyanine counterpart of this phthalocyanine showed a singlet oxygen quantum yield of 0.92 in the same solution [58]. The singlet oxygen quantum yield of the water-soluble asymmetric zinc (II) phthalocyanine compound containing six thiophene moieties was found as 0.81 in H_2_O [59]., Moreover, the axially silicon (IV) phthalocyanine compound bearing bis-benzimidazole moieties showed acceptable singlet oxygen quantum yield in aqueous solution (Φ_Δ_ = 0.78) [60]. These compounds can be candidates for photosensitizers in PDT applications due to their high singlet oxygen yields in water.

## 5. In vitro and in vivo biological applications of water-soluble phthalocyanines

The peripheral or nonperipheral positions and the central atom of the phthalocyanine ring can be made of these compounds as potential photosensitizers for biological and medical research areas [34]. Phthalocyanines are of great interest as photosensitizers for the treatment of malignant tumors in PDT. The therapeutic effects of these compounds are based on the formation of singlet oxygen (^1^O_2_) and other reactive oxygen species (ROS) formed upon light activation which is more uptake in malignant cells than in nonmalignant cells [61].

Water-soluble sulfonated aluminum phthalocyanine (Photosens), a phthalocyanine used in clinical tests, is investigated for the treatment of many cancer types, such as skin, breast, lung oropharyngeal, neck, larynx, and cervical cancers. Zinc phthalocyanine compound encapsulated in liposomes made from palmitoyl-oleoyl-phosphatidylcholine (POPC) and dioleoylphosphatidylserine (DOPS) is used for the treatment of upper aerodigestive tract carcinoma. Another di-sulphonic-di-phthalimidomethyl zinc phthalocyanine-based Cremophor EL formulation was tested for skin or esophageal cancer treatment. Silicone-based phthalocyanine formulations that dissolve in propylene glycol or Cremophor EL with ethanol and reach clinical tests for various skin diseases and cancers have also been developed [62]. Researchers proceed to study the development of water-soluble phthalocyanine compounds with photosensitizing properties superior to commercial compounds.

In vitro and in vivo studies of ionic and nonionic phthalocyanine compounds containing different groups on varied cell lines are given in Tables 2 and 3, respectively. The effects of groups on the phthalocyanine ring can be examined by looking at these tables.

## 6. Conclusion

PDT is a successful treatment modality that allows the destruction of cancerous and malignant tumors, resulting in selective photodynamic destruction. Since PDT compounds are activated by light of a specific wavelength that corresponds to the absorption band with the lowest energy, they are commonly referred to as photosensitizers. These photosensitizers produce cytotoxic substances known as reactive oxygen species, which destroy various intracellular structures and biologically important macromolecules, ultimately causing the death of cancerous tissue. Many photosensitizers, particularly those used on people, have low water solubility due to their tendency to aggregate. In PDT, photosensitizers become inactive due to accumulation and limited water solubility, greatly reducing their ability to perform in vivo. Therefore, it is very important to synthesize water-soluble phthalocyanine compounds, which have ideal photosensitizers in PDT applications. The water solubility of the phthalocyanine compounds increases their usability in various biological, medical, or environmental applications. Therefore, within the scope of this review, the results obtained from photophysical, photochemical, in vitro, and in vivo studies of water-soluble phthalocyanine compounds synthesized in the last five years are given in tables. Since the synthesis of water-soluble photosensitizers is of great importance in PDT applications, further studies should focus on the development of better photosensitizers. This review will have positive contributions to future studies on phthalocyanine chemistry and their PDT applications as well as photochemistry.

## Figures and Tables

**Figure:**
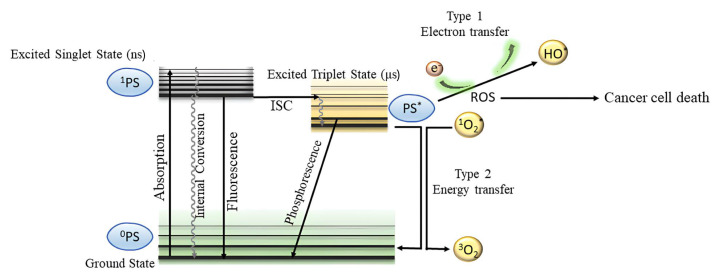
Type I and Type II mechanisms of photodynamic therapy.

**Table 1:** Photophysical and photochemical properties of ionic and nonionic water-soluble phthalocyanines.

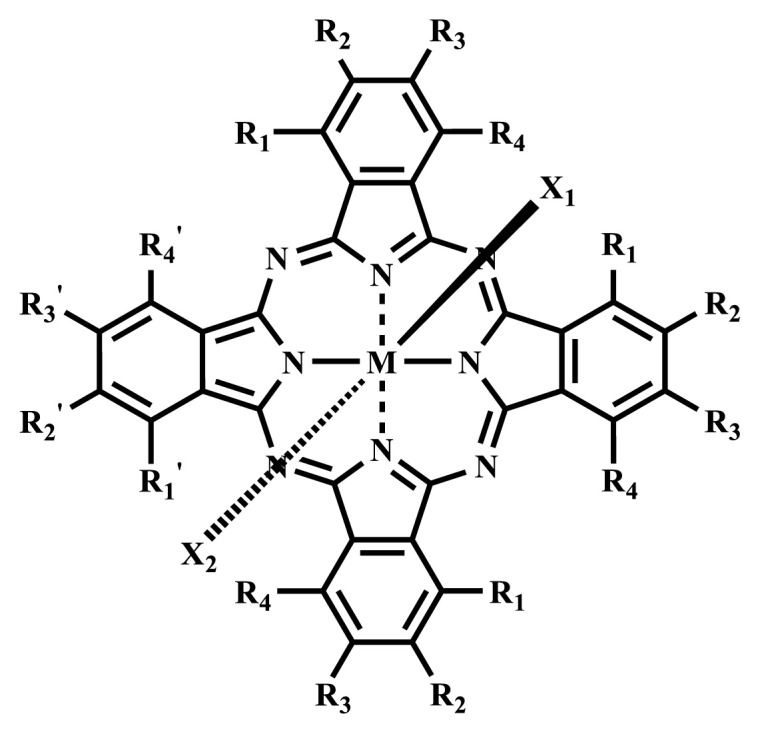							

Group	Metal	Solvent	λ_abs_ (nm)	τ_F_ (ns)	Φ_F_	Φ_Δ_	Ref.
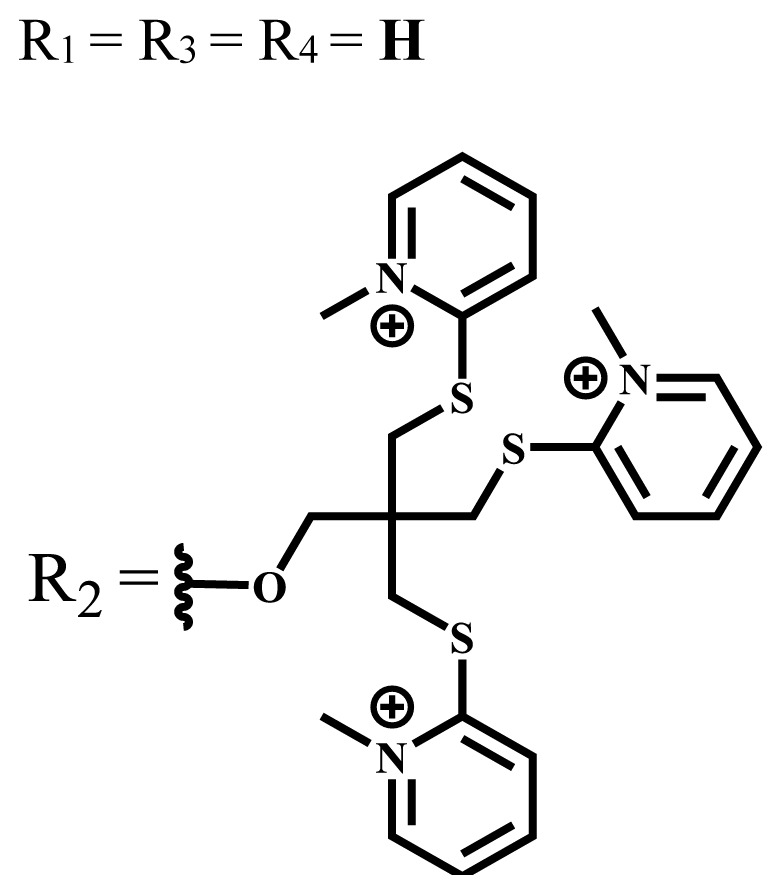	Zn	H_2_O	688	2.86	0.11	0.27	[36]

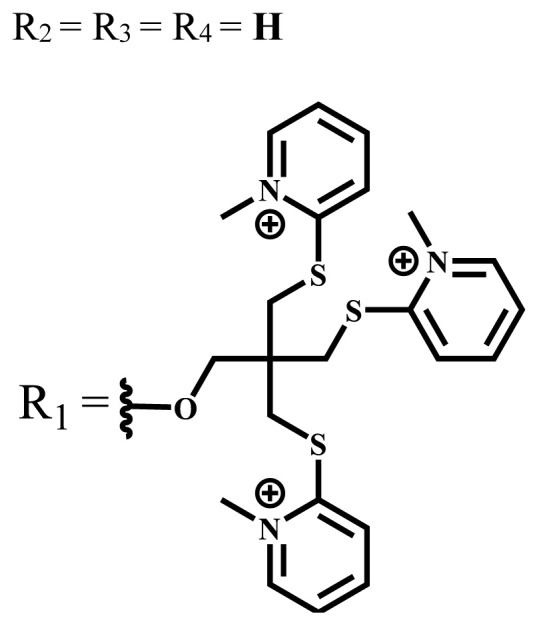	Zn	H_2_O	706	1.76	0.08	0.23	[36]

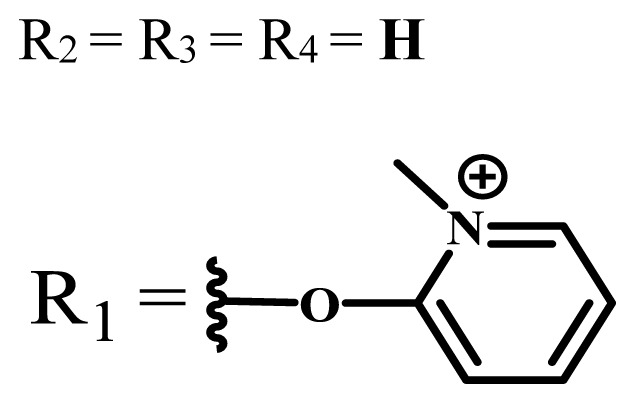	Pd	H_2_O	657	-	-	0.26	[6]
		H_2_O + TX^a^	666			0.46	
	Ni	H_2_O	668			0.01	
		H_2_O + TX^a^	679			0.02	

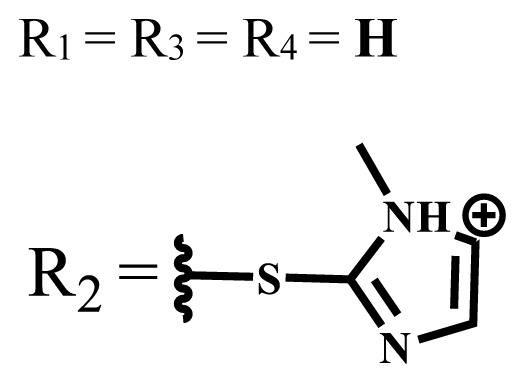	H_2_	H_2_O	604	-	-	0.24	[32]
	Zn		636			0.32	
	Ga		687			0.40	

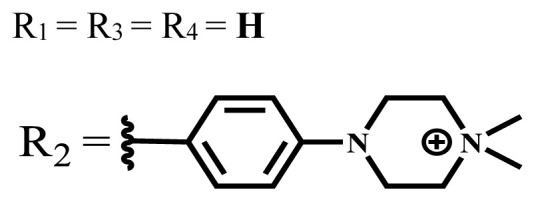	Zn	H_2_O + TX^a^	694	2.69	0.38	0.16	[63]

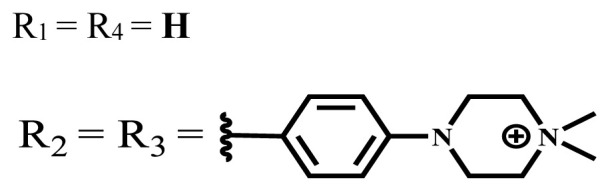	Zn	H_2_O + TX^a^	692	2.99	0.36	0.15	[63]

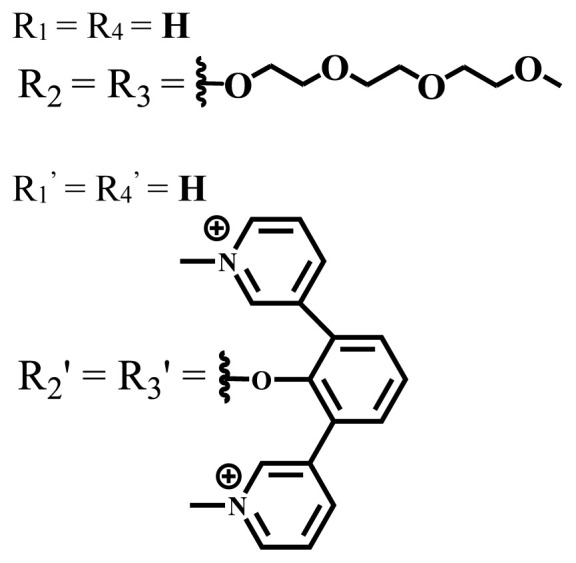	Zn	DMSO^b^	685	-	0.09	0.44	[64]

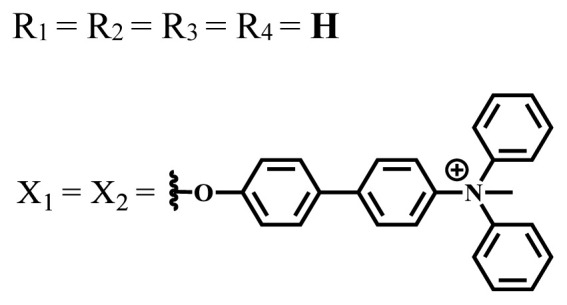	Si	H_2_O	691	4.46	0.17	0.26	[65]

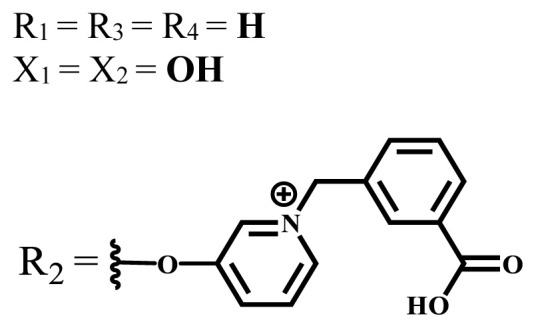	Si	H_2_O	679	-	0.12	0.03	[66]

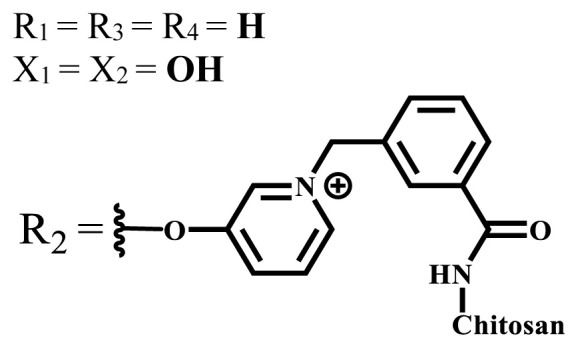	Si	H_2_O	679	-	0.10	0.27	[66]

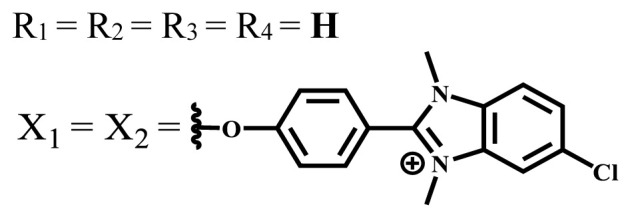	Si	Aqueous solution	691	1.12	0.02	0.78	[60]

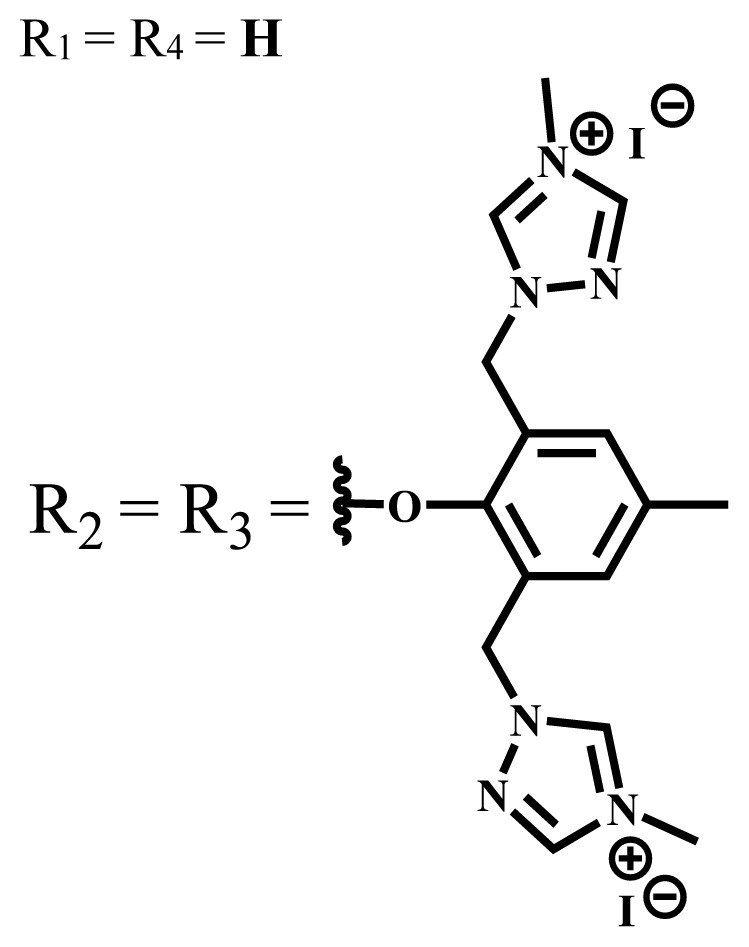	In	H_2_O	684	0.50 2.61	0.06	0.43 (D_2_O)	[67]

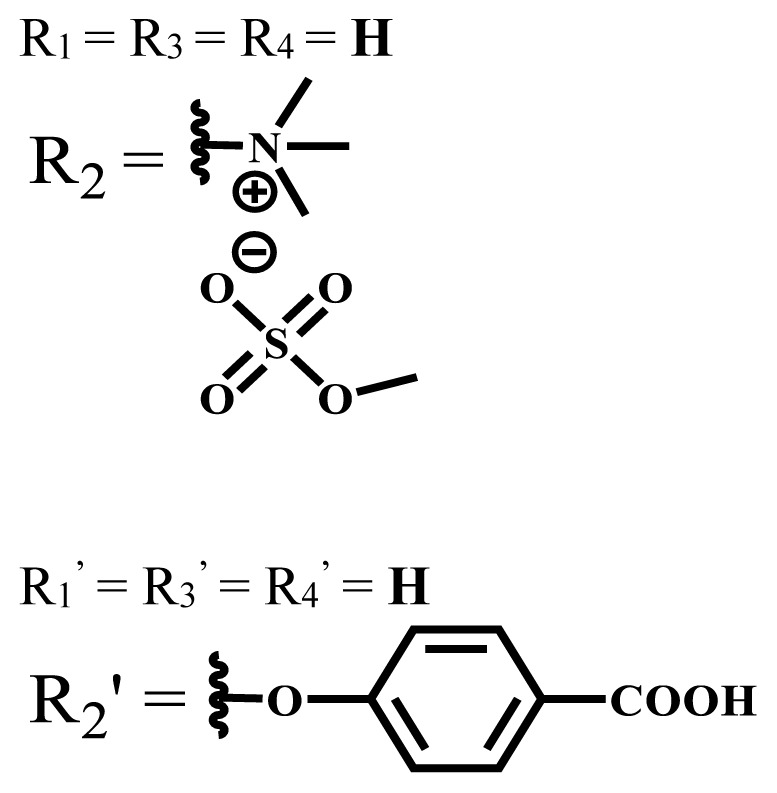	Zn	DMSO^b^	672	2.70	0.13	0.76	[68]

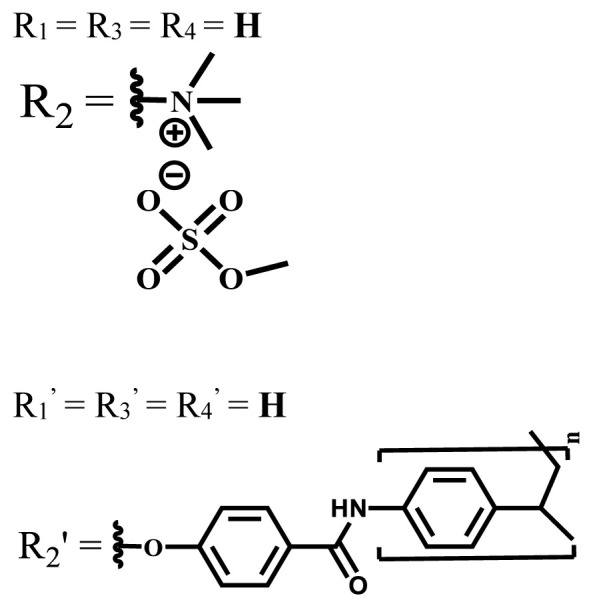	Zn	DMSO^b^	678	-	-	0.27 (H_2_O)	[68]

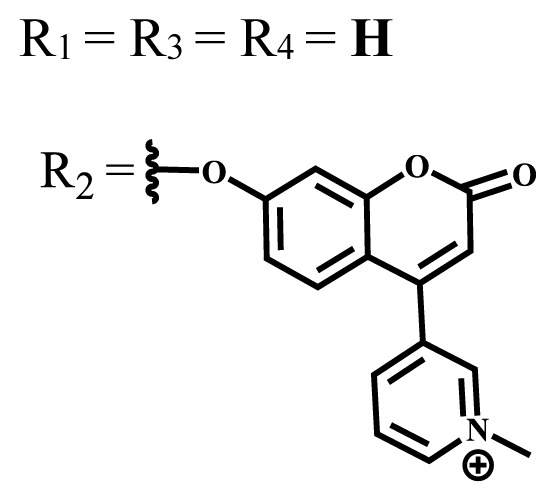	Zn	H_2_O	648	-	-	0.01	[58]
		H_2_O + TX^a^	679	1.29	0.06	0.15	
	In	H_2_O	689, 653	-	-	0.48	
		H_2_O + TX^a^	693	0.05	0.04	0.93	
	Mg	H_2_O	600	-	-	0.09	
		H_2_O + TX^a^	699	1.93	0.08	0.12	

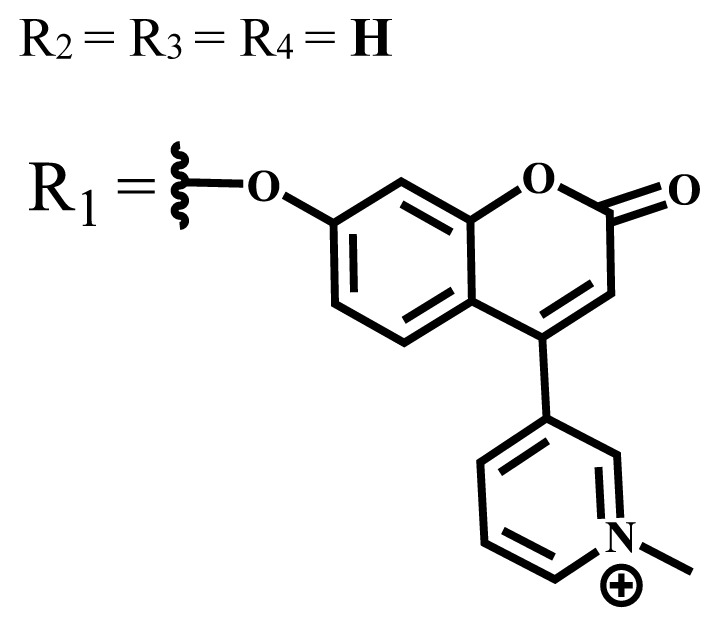	Zn	H_2_O	688, 650	-	-	0.06	[43]
		H_2_O + TX^a^	692	0.67	0.06	0.92	
	In	H_2_O	704, 650	-	-	0.08	
		H_2_O + TX^a^	703	0.03	0.02	0.41	
	Mg	H_2_O	694, 643	-	-	0.10	
		H_2_O + TX^a^	685	0.93	0.11	0.14	

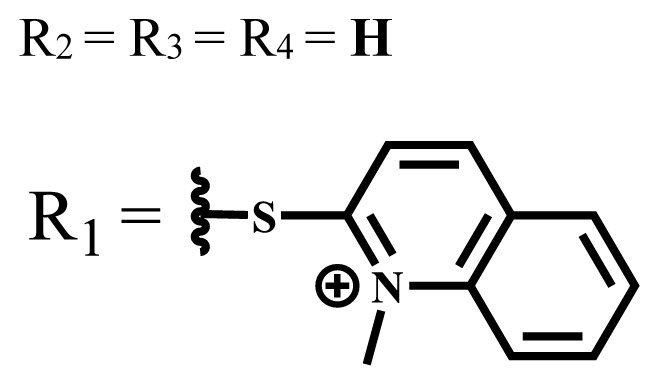	In	DMSO^b^	700		0.063	0.66	[69]
		H_2_O	654, 703	-	0.020	0.17	
		H_2_O + TX^a^	700		0.016	0.42	

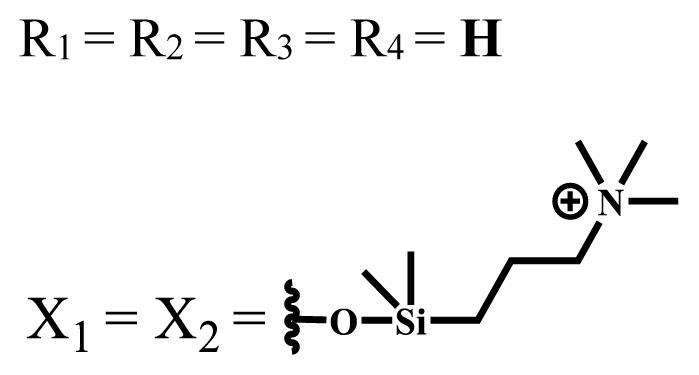	Si	H_2_O	676	-	0.15	0.44	[70]

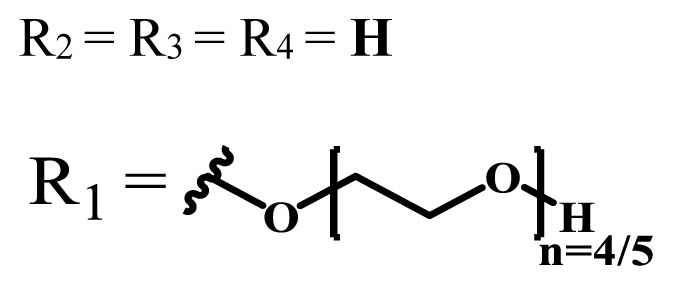	H_2_	DMSO^b^	704, 672		0.22	0.13 (H_2_O)	[71]
	Zn		682	-	0.18	0.05 (H_2_O)	
	Mg		680		0.29	0.04 (H_2_O)	

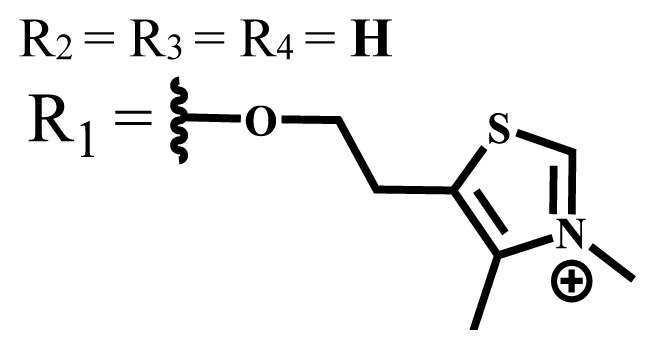	Zn	DMSO	704	1.84	0.072	0.85	[72]
		PBS^c^	703	1.58	0.1	0.58	

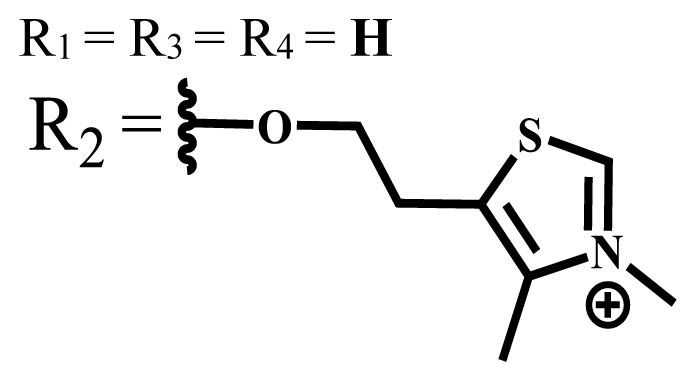	Zn	DMSO^b^	683	2.67	0.093	0.82	[72]
		PBS^c^	685, 647	2.30	0.195	0.80	

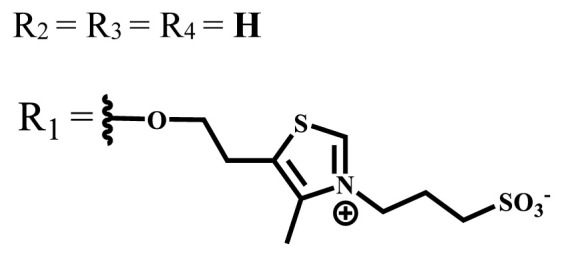	Zn	DMSO^b^	703	2.91	0.059	0.42	[72]
		PBS^c^	703	2.76	0.16	0.29	

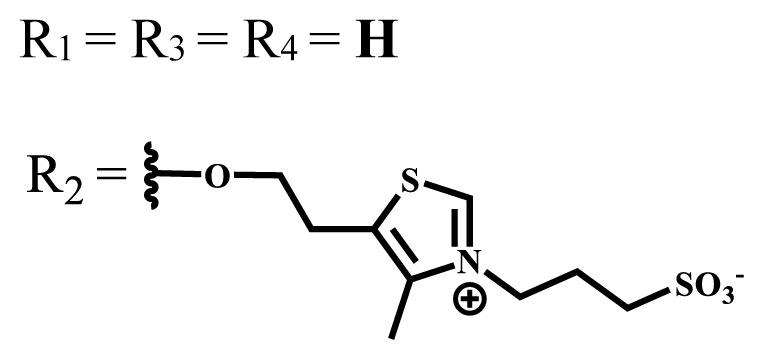	Zn	DMSO^b^	683	3.28	0.075	0.50	[72]
		PBS^c^	683, 631	0.679	0.023	0.12	

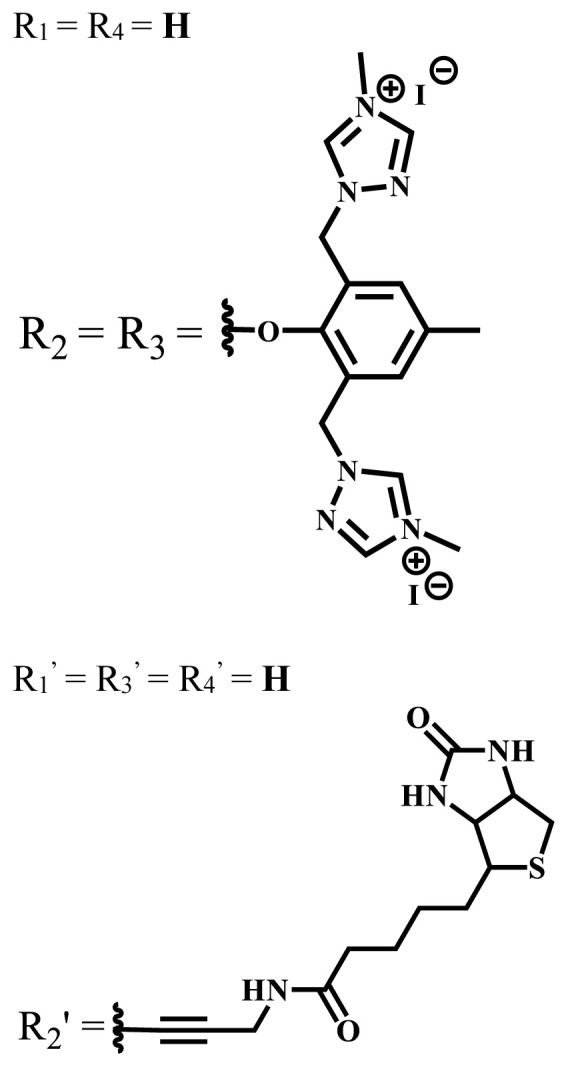	Zn	H_2_O	692	-	-	0.01	[73]

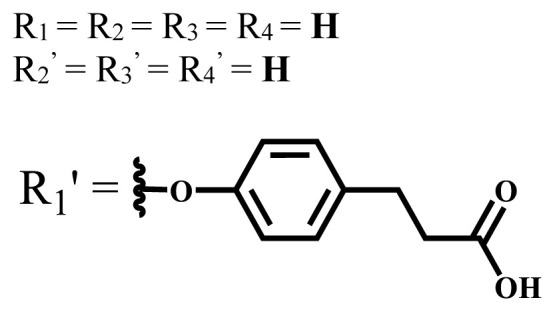	Zn	H_2_O	676	-	0	1.09	[74]

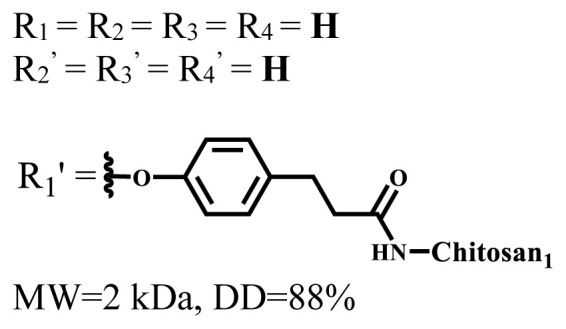	Zn	H_2_O	678	-	10.66 × 10^−3^	5.84 × 10^−3^	[74]

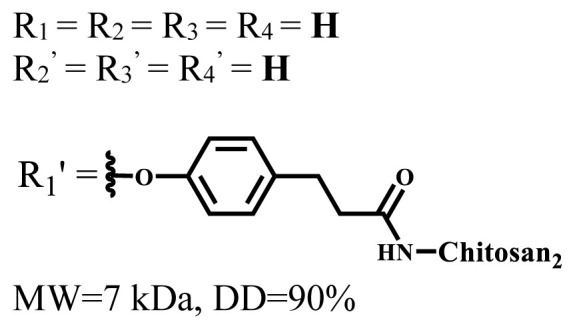	Zn	H_2_O	678	-	6.92 × 10^−3^	1.50 × 10^−3^	[74]

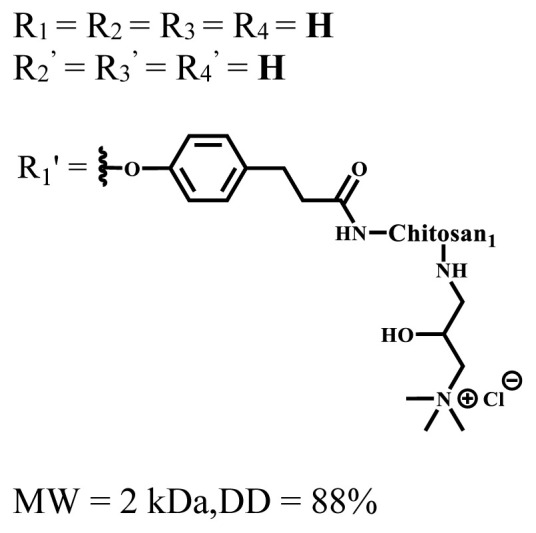	Zn	H_2_O	680	-	31.51 × 10^−3^	14.91 × 10^−3^	[74]

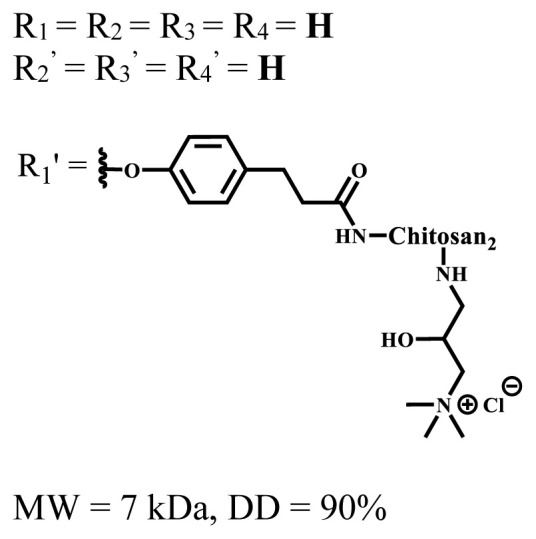	Zn	H_2_O	678	-	15.43 × 10^−3^	6.43 × 10^−3^	[74]

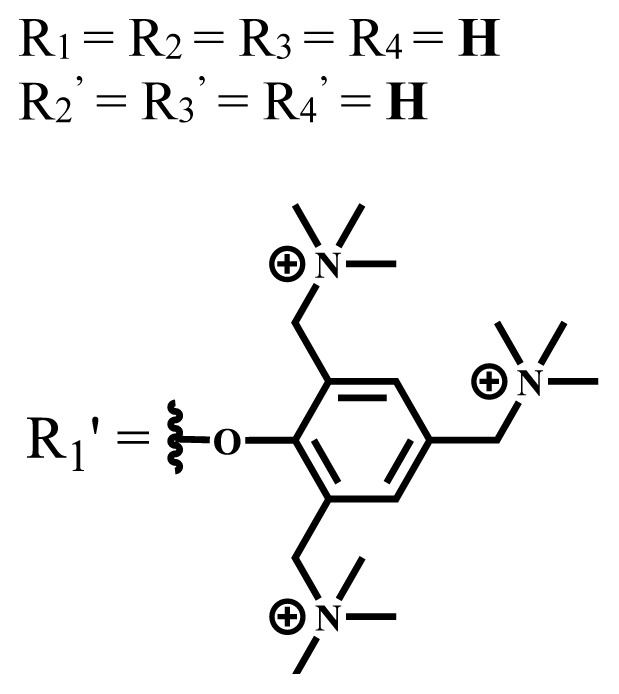	Zn	DMF^d^	682	-	0.20	0.66 (DMF) 0.61 (H_2_O)	[59]

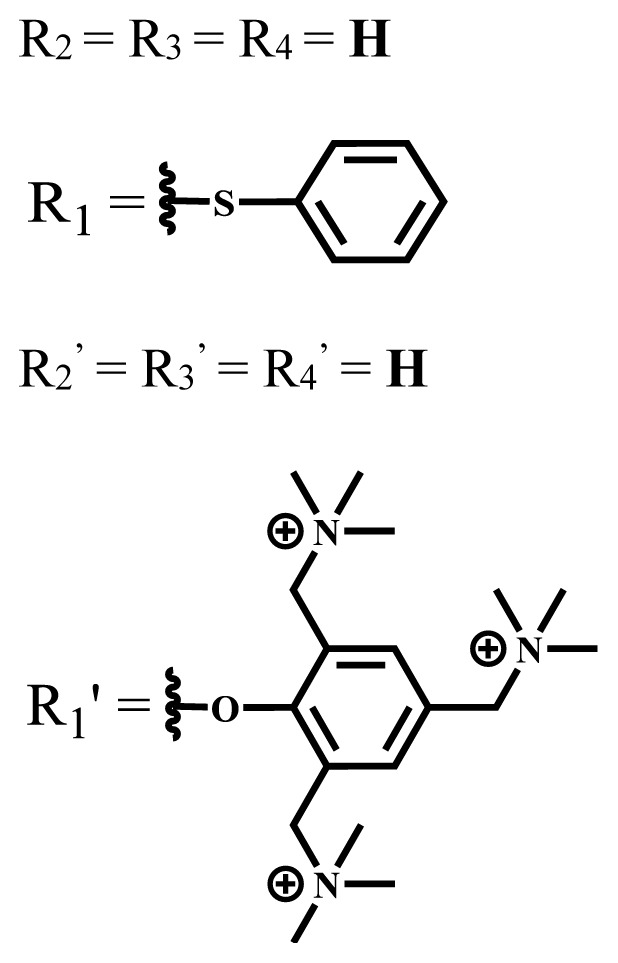	Zn	DMF^d^	705	-	0.06	0.72 (DMF) 0.76 (H_2_O)	[59]

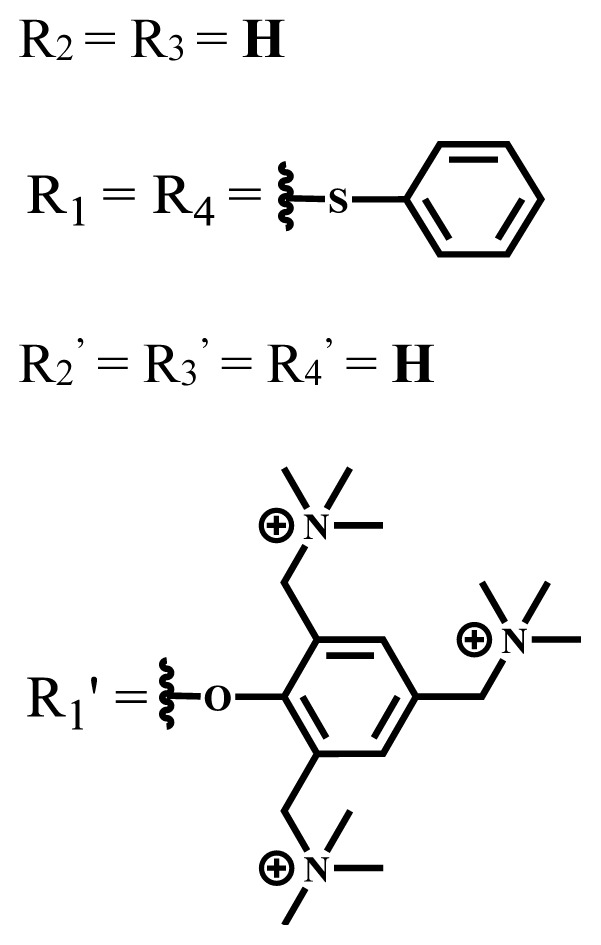	Zn	DMF^d^	758	-	0.02	0.89 (DMF) 0.81 (H_2_O)	[59]

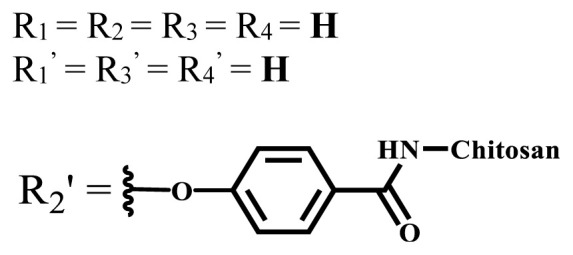	Zn	DMF^d^	671	-	0.26	0.35 (H_2_O)	[75]

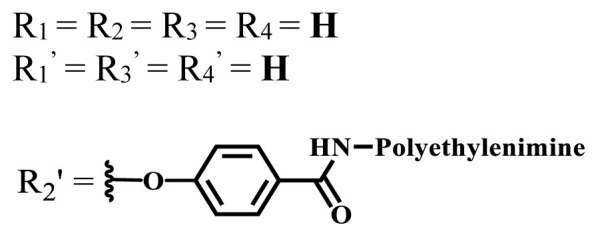	Zn	DMF^d^	670	-	0.30	0.31 (H_2_O)	[75]

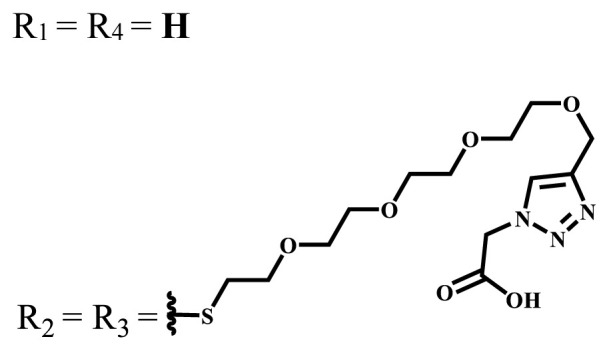	Zn	H_2_O	701	-		0.34	[76]

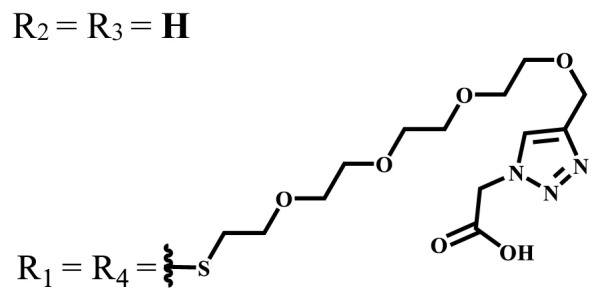	Zn	H_2_O	776		-	0.11	[76]

a TX: Triton X-100

b DMSO: Dimethyl sulfoxide

c PBS: Phosphate-buffered saline

d DMF: *N*,*N*-Dimethylformamide

**Table 2:** In vitro studies of ionic and nonionic water-soluble phthalocyanines.

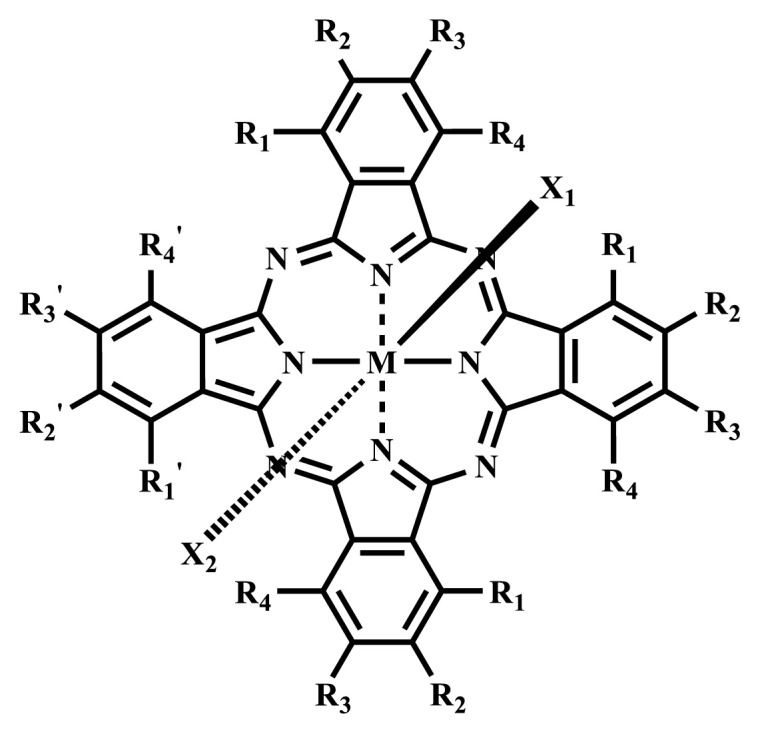									

Group	Metal	Solvent	λ_abs_ (nm)	Φ_F_	Φ_Δ_	Cell type	Dark toxicity	Light IC_50_	Ref.
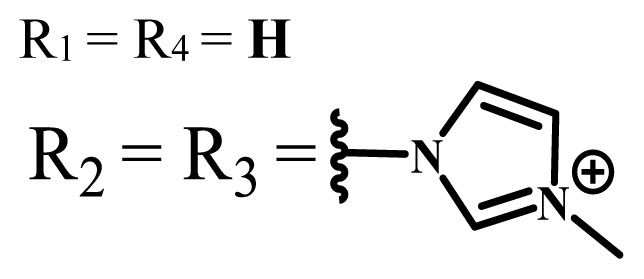	Zn	PBS	677	-	-	B16F10^a^	Slightly toxic at 0.1 mM	5.4 μM	[77]

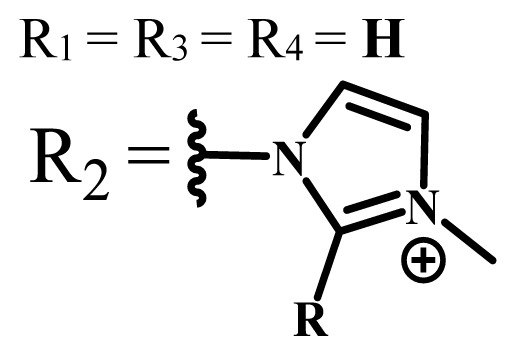	H_2_ R = **H**	DMSO	678	-	0.10	MDA-MB-231^b^ MCF-7^b^ A125^c^	Nontoxic	-	[78]
	Zn R = **H**		678		0.89	A431^c^ HaCat^d^			
	Zn R = **C_2_H_5_**		675		0.96	SW-480^d^ DU145^e^ BPH-1^e^			

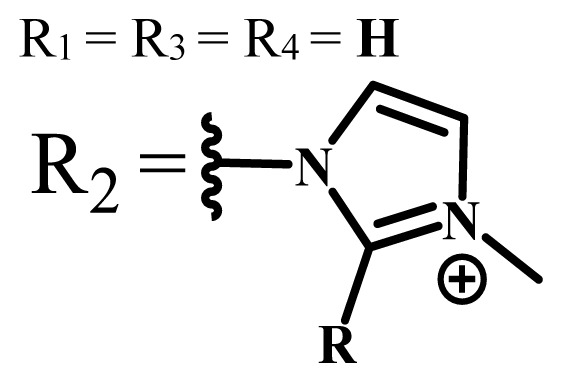	Fe R = **H**	DMSO	660	**-**	-	MDA-MB-231^b^ MCF-7^b^ A125^c^ A431^c^ HaCat^d^ SW-480^d^ DU145^e^ BPH-1^e^	Nontoxic	-	[79]
	Fe R = **C_2_H_5_**		634		-				
	Mg R = **H**		674		0.30				
	Mg R = **C_2_H_5_**		678		0.28				
	Mn R = **C_2_H_5_**		712		0.006				

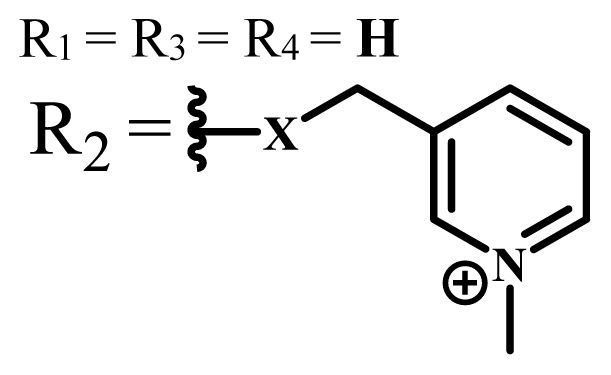	Zn X = **O**	DMSO	681.5	0.18	0.62	CT26^f^	Toxic	-	[80]
	Zn X = **S**		691.5	0.11	0.58		Nontoxic	1.40 μM	
	Zn X = **Se**		690.5	0.08	0.61		Nontoxic	8.50 μM	

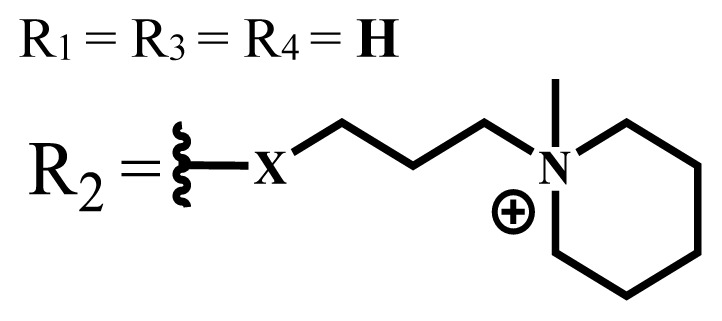	Zn X = **O**	DMSO	682	0.13	0.68	CT26^f^	Toxic	-	[80]
	Zn X = **S**		692	0.08	0.64		Nontoxic	2.20 μM	
	Zn X = **Se**		692.5	0.07	0.69		Nontoxic	3.50 μM	

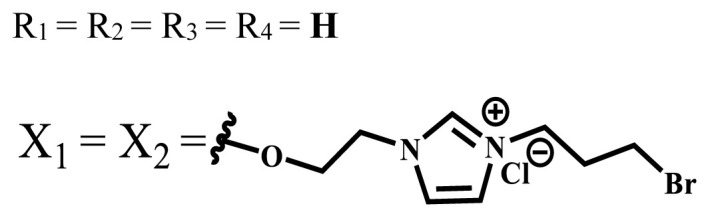	Si	H_2_O	683	-	0.12	HO-8910^g^	Almost no	2.0 μM	[81]

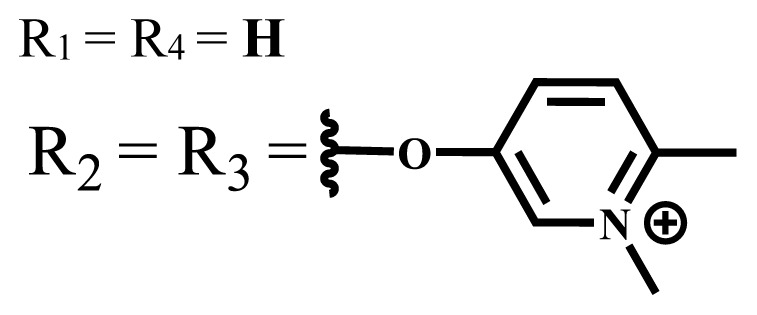	Ga	DMSO	681	0.12	4.4%	Hep2^h^	Nontoxic	> 100 μM	[82]
		PBS	679	0.14	0.029%				
	Zn	DMSO	678	0.15	5.3%			5.3 μM	
		PBS	673	-	0.009%				

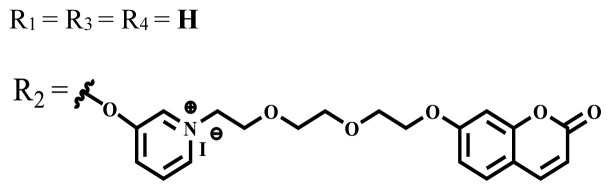	Zn	H_2_O	683	0.17	0.52	HepG2^i^	Nontoxic at 1 μM	-	[83]

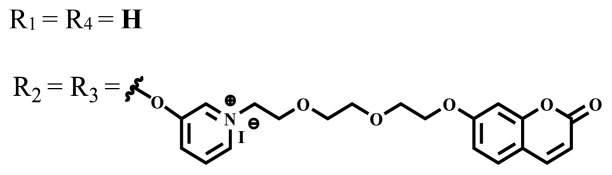	Zn	H_2_O	681	0.15	0.64	HepG2^i^	Nontoxic at 1 μM	-	[83]

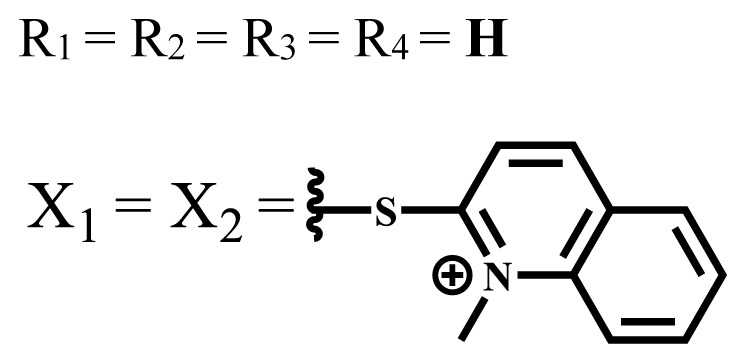	Si	H_2_O	673 (DMSO)	-	0.29	PC3^j^	-	-	[84]

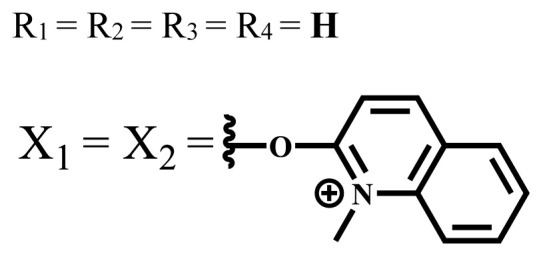	Si	H_2_O	676 (DMSO)	0.13	0.20	PC3^j^	-	-	[84]

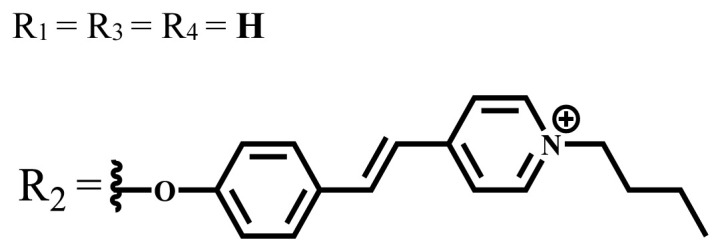	Zn	DMSO	683	0.03	0.62	MCF-7^k^	Negligible	8.2 μM	[85]

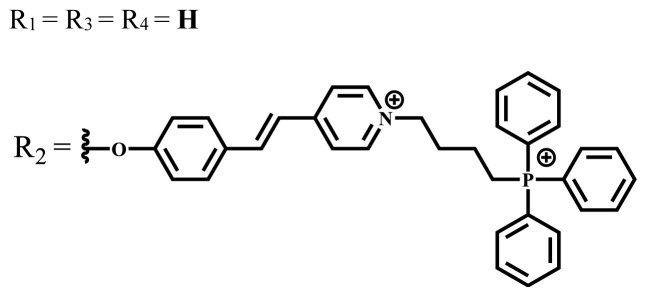	Zn	DMSO	684	0.06	0.53	MCF-7^k^	Negligible	4.9 μM	[85]

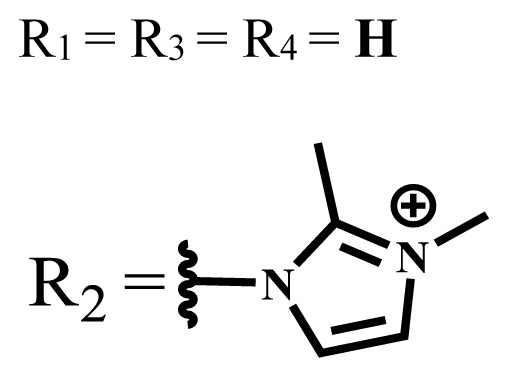	Zn	DMSO	675	0.20	0.42	MCF 7^k^	85% viable cells at concentration 80 μM	40% viable cells at 40 μM and 80 μM	[86]

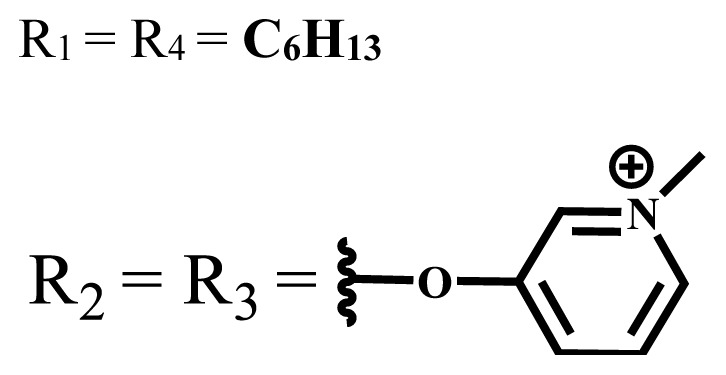	Zn	DMSO	725	0.06	0.67	MCF 7^k^	85% viable cells at concentration 80 μM	~ 55% viable cells at 40 μM and 80 μM	[86]

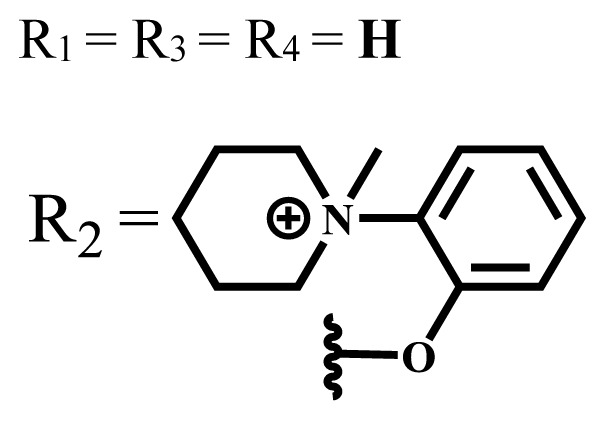	Zn	DMSO	681	-	0.76	HCT-116^l^	-	-	[87]

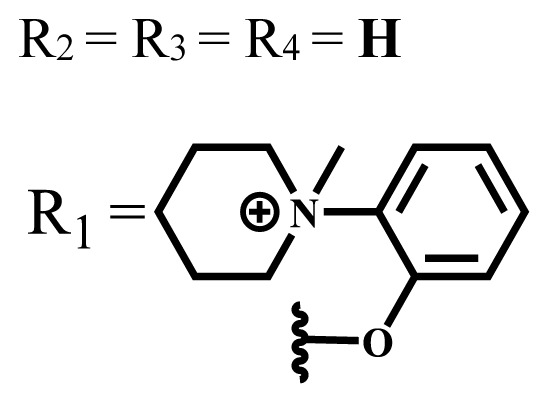	Zn	DMSO	701	-	0.78	HCT-116^l^	Low cytotoxic	**-**	[87]
						A549^m^	Toxic at 1, 5, and 10 μM	**-**	

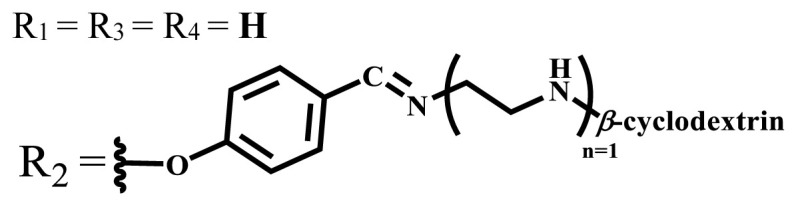	Zn	DMSO	695	-	0.87 (DMSO)	MDA-MB- 231^b^	Negligible	Cancer cells decreased to 27%	[88]

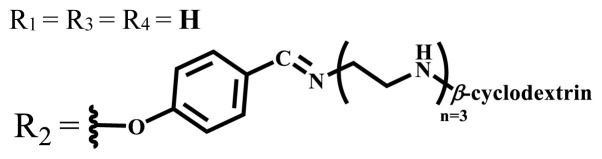	Zn	DMSO	700	-	0.95 (DMSO)	MDA-MB-231^b^	Negligible	Cancer cells decreased to 19%	[88]

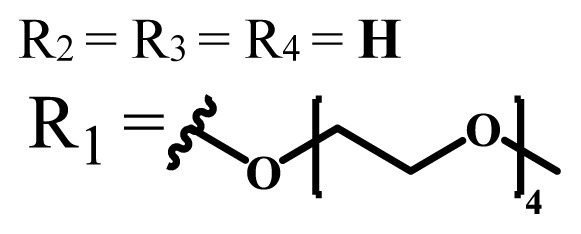	H_2_	DMSO	701, 727,	0.13	0.21	A253, FaDu^n^	Modest	-	[89]
	Zn		704	0.15	0.73	HT29^o^	Nontoxic		

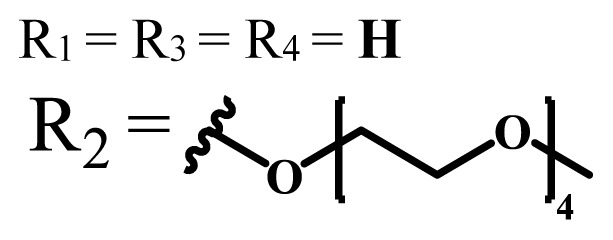	H_2_	DMSO	674, 706,	0.12	0.12	A253, FaDu^n^	Modest	-	[89]
	Zn		683	0.15	0.70	HT29^o^	Nontoxic		

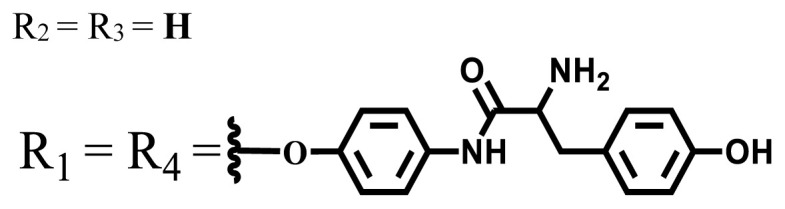	Zn	DMSO	680, 613	0.041	0.38	MDA-MB-231^b^ MCF-7^k^	Nontoxic	-	[90]

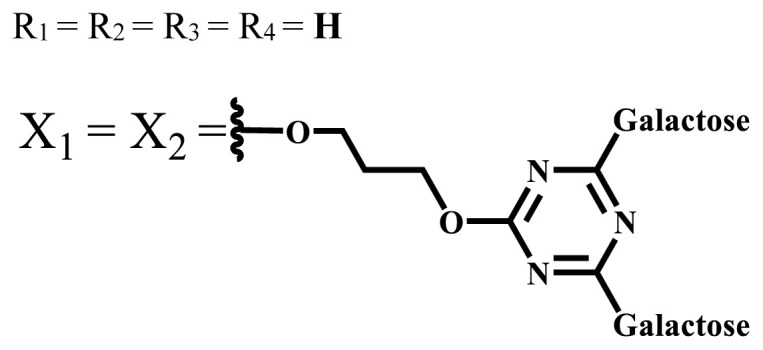	Si	DMF	690 PBS	0.38	Higher ability	UM-UC-3^p^	Not significant	-	[91]

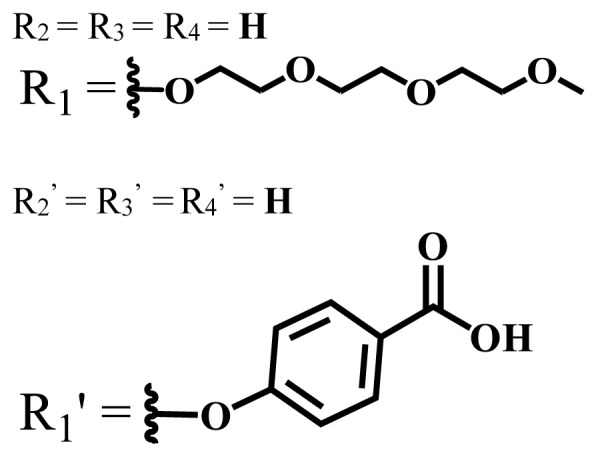	Zn	DMSO	696	-	0.49 MeOH	HeLa^r^	14.7 μM	0.426 μM	[92]
						A2780^s^	31 μM	0.211 μM	
						A2780 / CP70^t^	69.5 μM	1.2 μM	
						MRC-5^u^	48.1 μM	1.11 μM	

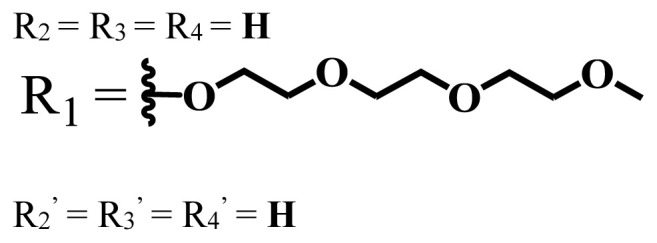	Zn	DMSO	694	-	0.84 MeOH	HeLa^r^	5.2 μM	0.009 μM	[92]
						A2780^s^	44 μM	0.018 μM	
						A2780/CP70^t^	117.5 μM	0.157 μM	
						MRC-5^u^	12.8 μM	0.019 μM	

a B16F10: Murine melanoma cancer cell

b MDA-MB-231 and MCF-7: Human breast cancer cell

c A125 and A431: Lung cancer cell

d HaCat and SW-480: Colon cancer cell

e DU145 and BPH-1: Prostate cancer cell

f CT26: Colon carcinoma cell

g HO-8910: Human ovarian cancer cell

h Hep2: Human carcinoma cell

i HepG2: Human hepatocarcinoma cell

j PC3: Human prostate cancer cell

k MCF-7: Breast cancer cells

l HCT-116: Human colorectal carcinoma cell

m A549: Human lung adenocarcinoma cell

n A253, FaDu: Head and neck cancer cell

o HT29: Colon cancer cell

p UM-UC-3: Human bladder cancer cell

r HeLa: Human cervical cancer cell

s A2780: Cisplatin-sensitive human ovarian carcinoma cell

t A2780/CP70: Cisplatin-resistant ovarian endometrioid adenocarcinoma cell

u MRC-5: Noncancerous fibroblast cell

**Table 3:** In vivo studies of ionic and nonionic water-soluble phthalocyanines.

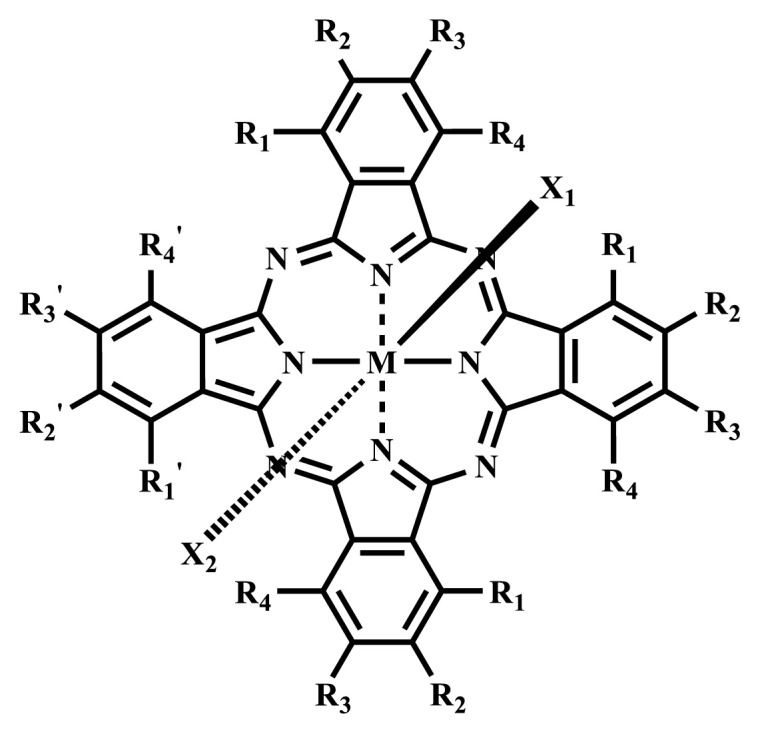							

Group	Metal	Solvent	λ_abs_ (nm)	Φ_F_	Φ_Δ_	In vitro and in vivo assays	Ref.
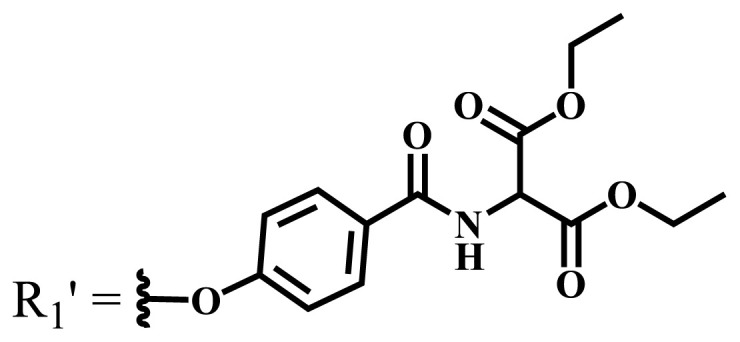	H_2_	H_2_O	730	-	-	HeLa cells Low cytotoxicity 4T1 tumor bearing BALB/c mice mammary carcinoma model	[93]
	Zn				-		
	Cu				32.3%		

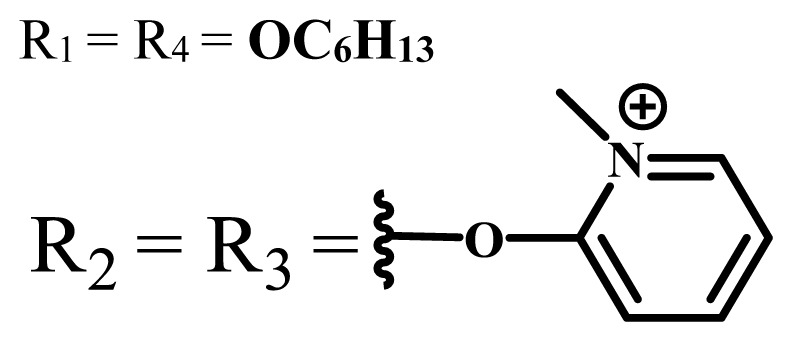	Zn	DMF	712	-	Generate ROS	HepG2 No significant dark toxicity IC_50_ = 11 μM H22 tumor-bearing mice	[94]

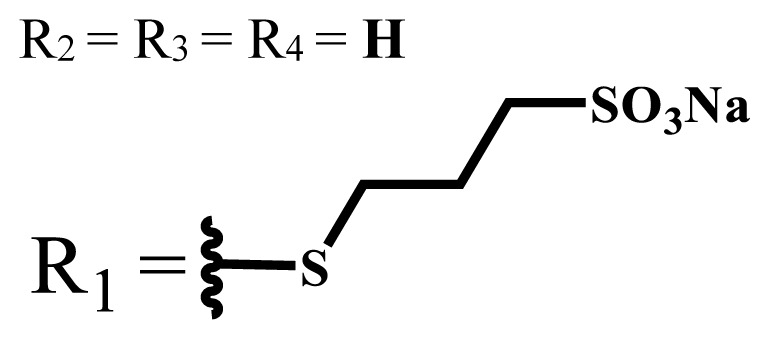	Si	DMF	680	0.31	0.14	HepG2 Noncytotoxic IC_50_ = 0.023 μM Mice bearing H22 murine hepatocellular tumor	[95]

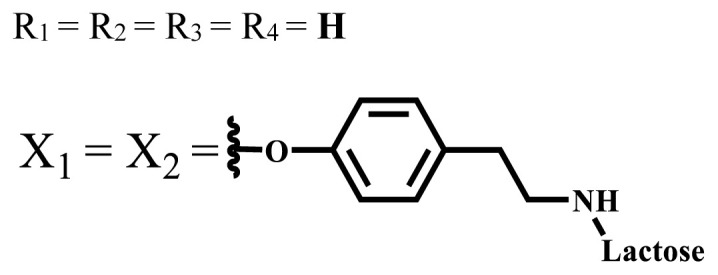	Zn	PBS	711, 671	0.003	0.41 (D_2_O)	MDA-MB-231 and MCF-7 Not toxic in dark IC_50_ > 100 μM Mice with head and neck squamous carcinoma cells	[96]

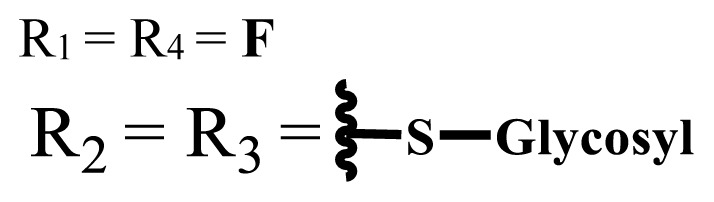	Zn	DMSO	681	-	0.56	A431 IC_50_ = 380 nmol/L A549 IC_50_ = 220 nmol/L MCF-7 IC_50_ = 240 nmol/L PC-3 IC_50_ = 280 nmol/L Noncytotoxic in the dark Human A431 tumor bearing BALB/c nude mice	[97]

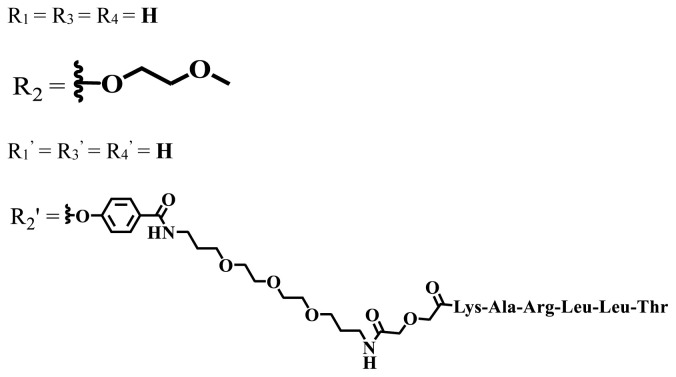	Zn	DMSO	681	-	0.60	A431 IC_50_ = 240 nmol/L A549 IC_50_ = 740 nmol/L MCF-7 IC_50_ = 130 nmol/L PC-3 IC_50_ = 330 nmol/L Noncytotoxic in the dark Human A431 tumor bearing BALB/c nude mice	[97]

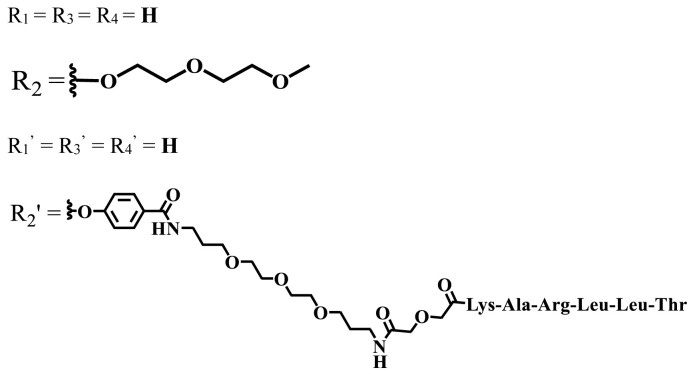	Zn	DMSO	680	-	0.54	A431 IC_50_ = 220 nmol/L A549 IC_50_ = 170 nmol/L MCF-7 IC_50_ = 520 nmol/L PC-3 IC_50_ = 650 nmol/L Noncytotoxic in the dark Human A431 tumor bearing BALB/c nude mice	[97]

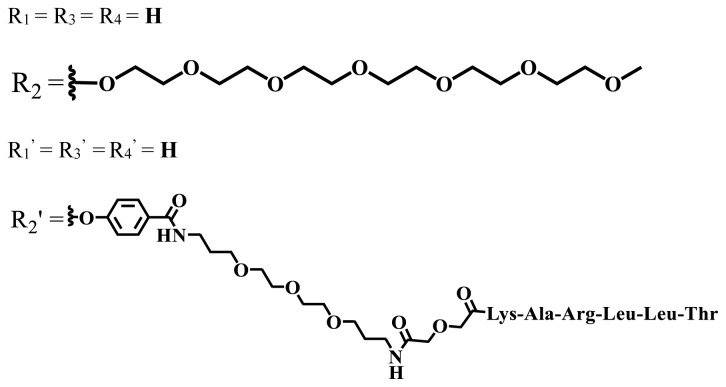	Zn	DMSO	680	-	0.49	A431 IC_50_ = 190 nmol / L A549 IC_50_ = 320 nmol / L MCF-7 IC_50_ = 230 nmol / L PC-3 IC_50_ = 440 nmol / L Noncytotoxic in the dark Human A431 tumor bearing BALB/c nude mice	[97]

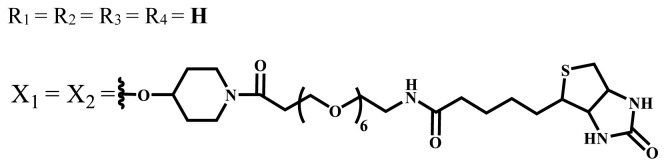	Si	DMF	675	0.35	0.42	HeLa Almost noncytotoxic 123 nM Tumor-bearing mice	[98]
